# The role of emotional intelligence in shaping pre-service teachers’ cultural beliefs

**DOI:** 10.1371/journal.pone.0331282

**Published:** 2025-09-04

**Authors:** Sotiria Varis, Asko Tolvanen, Myrto Kyriazopoulou, Anu Laine, Katariina Waltzer, Anna Widlund, Lisa E. Kim, Robert Klassen, Riitta-Leena Metsäpelto

**Affiliations:** 1 Department of Teacher Education, Faculty of Education and Psychology, University of Jyväskylä, Jyväskylä, Finland; 2 Faculty of Education and Psychology, University of Jyväskylä, Jyväskylä, Finland; 3 Faculty of Educational Sciences, University of Helsinki, Helsinki, Finland; 4 School of Educational Sciences and Psychology, University of Eastern Finland, Joensuu, Finland; 5 Faculty of Education and Welfare Studies, Åbo Akademi University, Turku, Finland; 6 School of Psychology, University of Sydney, Sydney, Australia; 7 Department of Education, University of Oxford, Oxford, England; University of Valencia: Universitat de Valencia, SPAIN

## Abstract

Cultural beliefs and emotions have become recognised aspects of the teaching profession. This study examined the association between emotional intelligence (EI) and cultural beliefs using self-reported survey data collected from pre-service teachers at the beginning of their studies (N = 777). These pre-service teachers entered teacher education in 2021 and were studying in five different Finnish universities that geographically represent four regions in Finland. The results from the confirmatory factor analysis and the multiple linear regression suggested the four dimensions of EI positively predict the latent factors of both multicultural and egalitarian beliefs. The relationships between the constructs exhibited moderate effect sizes, with slightly stronger associations found for multicultural beliefs (β = 0.38) compared to egalitarian ones (β = 0.26). Moreover, the ‘using emotions’ dimension of EI significantly and positively predicted pre-service teachers’ multicultural beliefs; the use of emotions to guide thinking significantly predicted beliefs favouring cultural diversity. The study provides further empirical evidence for the influence of emotions on beliefs, and stresses the need to address topics pertaining to democratic education more explicitly and consistently in pre-service teacher education.

## Introduction

Due to increasing ethnic and cultural diversity globally [1], cultural beliefs have become a recognised aspect of teachers’ professional competencies. Teachers’ beliefs act as filters that inform teachers’ pedagogical decision making and interpretation of professional experiences [2]. Cultural beliefs are therefore crucial to discussions on culturally responsive teaching and teacher development. Research suggests that teachers’ openness to cultural diversity affects teachers’ professional satisfaction and sense of efficacy, perceived warmth in teacher-student relationships, comfort when teaching in multicultural contexts, and negative or positive perception of students [3]. Research further shows that teachers’ multicultural beliefs may reduce prejudice [4]. This suggests multicultural perspectives are a promising component of teacher education, especially because pre-service teachers lack mastery experiences and report negative implicit attitudes towards ethnic minority students [5]. However, the significant implications of teachers’ commitment to non-discrimination and non-repression for teacher quality and teacher education alike draw attention to how culturally responsive dispositions are developed during pre-service teachers’ studies [6].

Beliefs are considered a major determinant of emotion, while emotions have a notorious influence on the content and strength of beliefs as well as their resistance to change [7]. Both beliefs and emotions are psychological processes organised in form and function by how individuals interpret their various environments, as well as by how individuals’ interpretations are reciprocally constituted by the culture within such environments [8]. Although emotions are now considered to be a central aspect of teachers’ professional lives, there are challenges to exploring the connection between teachers’ emotions and their cultural beliefs. Emotions are often overshadowed by cognitive constructs in educational literature on teacher knowledge, even though “beliefs *and emotions* come into play as teachers make decisions, act and reflect on the different purposes, methods and meanings of teaching” [9, p. 467]. Indeed, emotions significantly inform what beliefs we hold and how strongly we hold them [7], thus making their impact on cultural beliefs highly relevant to discussions about teacher education amidst changing classroom landscapes.

The earlier primacy of beliefs over emotions in educational literature has been amended in more recent research on teacher education and–particularly–second language teaching [10]. At the same time, however, a plethora of terms has been developed over the past thirty years to refer to teachers’ cultural beliefs (e.g., teachers’ beliefs about multiculturalism, teacher cultural models, teachers’ beliefs about cultural diversity, teachers’ views of cultural diversity, cultural beliefs, and teachers’ acculturation attitudes), without a generally accepted classification system [11]. Moreover, most of the studies on social, emotional, and intercultural competencies do not examine these competencies simultaneously and rely on self-reported survey data [12]. To examine such competencies holistically, this study draws on the concept of emotional intelligence [13] to address social and emotional competencies, and on the concept of cultural beliefs [4,14] to address intercultural competencies.

This study is situated within the context of Finnish teacher education. Teacher education in Finland differs from many other countries in that prospective teachers undergo a rigorous two-phase student selection process. This process evaluates applicants’ cognitive skills and suitability for teaching using matriculation examination scores, a source-based multiple-choice test, and multiple mini interviews. Among other competencies, the multiple mini interviews interviews typically assess applicants’ suitability in terms of socio-emotional skills and attitudes towards diversity. Knowing how applicants accepted into initial teacher education (ITE) self-report on such competencies early in their studies is important for two reasons. First, because it indicates whether procedures of student selection into ITE adequately assess the existing and future potential of its applicants. Such information would arguably be important to student selection procedures assessing similar competencies in different post-secondary education contexts [15]. Second, because it may inform ITE as to whether curricular elements should support the development of such competencies already at the beginning of pre-service teachers’ studies, especially concerning pre-service teachers reporting low scores on emotional intelligence or cultural beliefs.

This nationwide study examines such pre-service teachers’ competencies through first-year students’ self-reported cultural beliefs alongside emotional intelligence scores. The aim of this study is to statistically examine potential associations between the four branches of emotional intelligence (EI; perceiving emotions, using emotions, understanding emotions, and managing emotions) [16] and two aspects cultural beliefs (multicultural beliefs and egalitarian beliefs) [14].

Based on the rigorous student selection for Finnish ITE and the findings of the few studies jointly examining teachers’ EI and cultural beliefs or attitudes [17–19], we hypothesize that EI will be positively associated with cultural beliefs. That is, we anticipate that pre-service teachers who report highly on EI will score higher in cultural beliefs. Using survey data from five Finnish universities offering teacher education, we explored the following research question: What are the associations between first-year pre-service teachers’ EI and cultural beliefs? The following section elaborates on the key theoretical constructs used in this study.

## Theoretical framework

### Teachers’ cultural beliefs

Social-psychological research suggests that there are different beliefs that have different implications for social interaction [14]. In educational psychology, beliefs may be understood as teachers’ espoused suppositions, ideologies, commitments, attitudes, views, or models about students. More specifically, teachers’ cultural beliefs from a social-psychological standpoint are subjective claims and perspectives on intergroup ideologies related to culturally diverse school contexts [20].

Hachfeld et al. [14] explain two such distinguishable and conceptually independent beliefs, multiculturalism and egalitarianism. *Multicultural beliefs* are cultural beliefs which emphasise differences as enriching and something to be embraced. They additionally recognize the importance of different perspectives and beliefs due to individuals’ engagement with various socio-cultural contexts. *Egalitarian beliefs* are cultural beliefs which emphasise similarities and common grounds. They focus on equality and treating everyone similarly regardless of ethnic and cultural background. Egalitarian beliefs entail de-emphasizing differences and stressing similarities, which are two distinct aspects of colourblindness and influence the importance teachers ascribe to group memberships [20].

Based on Hammer et al.’s [21] conceptualization of orientations in intercultural sensitivity, we could argue that multicultural beliefs stem from more ethnorelative worldviews, whereby cultural difference is sought by accepting its importance, adapting one’s perspective to it, or integrating it into one’s understanding of identity. Egalitarian beliefs stem from more ethnocentric orientations, whereby cultural difference is avoided by denying or defending its existence, or by minimising its importance. In developing their own instrument to examine teachers’ beliefs about cultural diversity in the Spanish context, López López and Hinojosa Pareja [22] found that multicultural beliefs are favourable beliefs. Multicultural beliefs are, however, distinguishable from other approaches to cultural diversity because they are not based on an egalitarian conception of cultural diversity [22]. While not mutually exclusive, multicultural and egalitarian beliefs are distinct [20]. Despite their different foci, combining the two analytically separate concepts of multicultural and egalitarian beliefs contribute a pluralistic approach to understanding teachers’ attitudes and decision-making in educational contexts characterised by diversity.

Although teachers’ cultural beliefs are important to pedagogical decision-making and their students’ socio-academic adaptation at school [20], there is a lack of empirical results in educational research from surveys analytically examining distinct aspects of cultural beliefs [14]. Having such results is important when research on teachers’ multicultural and egalitarian beliefs suggests they have varying importance in teachers’ practice. Most quantitative empirical research on teachers’ cultural beliefs concerns participants’ self-reported proficiency to teach in culturally diverse classrooms, showing promising results for teachers advocating multicultural beliefs (e.g., particular enthusiasm for teaching immigrant students, decreased likelihood of holding negative ethnic prejudices, reported use of more effective problem-solving strategies, and willingness to adapt instructional practices to individual student needs) [20]. Although multicultural beliefs may “not necessarily imply teaching that transmits a true valuation of human diversity,” such beliefs are “an inadequate, but necessary, condition for ideal learning” within contexts of diversity, wherein “the lack of multicultural beliefs is more defining in the quality of teaching than [their] presence” [23, p. 859].

Quantitative empirical research has yielded inconclusive results for teachers advocating egalitarian beliefs [20]. Such research finds that egalitarian beliefs have no impact on teachers’ self-efficacy and enthusiasm, and no impact on students’ academic achievement and psychological school adjustment [20]. Other research shows that exposure to colourblind ideologies generates greater automatic racial bias, whereas exposure to multicultural ones may increase interracial harmony [4]. However, researchers also caution that treating all students equally by ignoring differences can lead to a lack of preparation for the challenges of a diverse classroom. Teachers being unable to adapt their teaching to diversity can lead to a lack of adequate support and accurate assessment for immigrant students’ performance, possibly resulting in systematic discrimination [1]. As seen in pre-service teachers, having an ethnic majority or ethnic minority background affects the implicit and explicit attitudes–including multicultural beliefs–one has toward students from ethnic minority groups [4]. Implicit attitudes inform pre-service teachers’ behaviour, judgments, and behavioural intentions in the classroom, even more so when pre-service teachers are unaware of the inﬂuence of such attitudes or when cognitive resources are limited [24]. These findings point to the importance of cultural contexts in shaping teachers’ cultural beliefs, and suggest that how teachers encounter, interpret, and respond to diversity is informed by the multicultural or egalitarian beliefs they hold [14].

Although cultural differences and contexts play a significant role in shaping our understanding, application, and evaluation of EI [8,25], there have been no quantitative studies to date examining teachers’ multicultural and egalitarian beliefs alongside pre- or in-service teachers’ EI. However, a handful of recent studies suggest there may be a connection between these constructs. Empirical evidence from in-service primary school teachers in Turkey indicates EI efficacy has a significant impact on teachers’ multicultural attitudes [17]. Empirical evidence from pre-service teachers in Oregon shows that inclusive dispositions, including EI and cultural competence, are significantly correlated with instructional choices for culturally and linguistically diverse students [18]. Moreover, research examining the associations between pre-service teachers’ personality traits and diversity beliefs suggests a significant correlation between diversity beliefs and Emotionality, which is a facet of Openness; the findings suggested that pre-service teachers scoring higher on Openness may benefit more from diversity-related educational efforts than lower-scoring peers [26]. The following section elaborates on EI in teacher education.

### Teachers’ emotional intelligence

Emotional intelligence (EI), a concept popularised in the 1990s [27], has been central to the field of educational psychology across levels of education. The Ability model of EI is one of the most prominent among EI theories and examines EI as a set of cognitive abilities [13]. The Ability model of EI posits that EI consists of the abilities to perceive, use, understand, and manage emotions in the self and others. ‘Perceiving emotions’ is the ability to accurately perceive and recognize emotions in oneself and others. It involves recognising facial expressions, vocal tones, and other nonverbal cues that convey emotions. ‘Using emotions’ to facilitate thinking is the ability to utilize emotions to enhance cognitive processes. It involves using emotions to aid problem-solving, decision-making, and creativity. ‘Understanding emotions’ focuses on comprehending the complexities of emotions, including the ability to label emotions accurately, understand the causes of emotions, and appreciate how emotions can evolve and blend together. ‘Managing emotions’ involves the ability to effectively regulate one’s emotions and the emotions of others. This entails using strategies for managing stress, calming oneself, and motivating oneself and others.

In an increasingly interconnected and emotionally loaded school, teachers’ EI is of central importance. Chang noted that “the habitual patterns in teachers’ judgments about student behaviour and other teaching tasks may contribute significantly to teachers’ repeated experience of unpleasant emotions and those emotions may eventually lead to burnout” [28, p. 193]. Research has shown that teachers’ EI has a protective role against burnout, and that it is positively associated with life satisfaction and reduced stress [29,30]. In addition, EI is particularly relevant and important as a basic competency for pre-service teachers, who are preparing to enter the reality of the teaching profession [31]. Pre-service teachers with high levels of EI have numerous benefits regarding their studies, such as course satisfaction and successful classroom teaching experiences [32].

From a theoretical standpoint, individuals with high levels of EI tend to be more social, have positive relationships, and are pro-active individuals with increased empathy [29,33,34]. Since the knowledge and understanding of emotional cues and their interpretation makes such individuals more skilled in interactions [16], it could be argued that in a multicultural context individuals with a higher ability to perceive and understand emotions would be more open and have more pro-multicultural attitudes. At the same time, teachers endorsing multicultural beliefs are more likely to actively acknowledge and respond to immigrant pupils’ socioemotional and learning needs, as well as sustain responsive and respectful relationships with them [20]. In contrast, teachers with egalitarian beliefs may promote positive intergroup relations but be less pedagogically adaptive and less responsive to immigrant pupils’ need for a distinct social identity [20]. EI abilities may thus be theoretically relevant to discussions about teachers’ willingness to acknowledge cultural diversity in their students and adapt instruction in a culturally responsive manner. For example, research in the Danish context suggests a positive association between primary school teachers’ EI and their multicultural attitudes [19]. However, no study so far has explored this association with pre-service teachers nor alongside multicultural and egalitarian beliefs.

## Methods

### Participants

The dataset used in this study originates from a project focusing on the admission process into teacher education programmes and on the development of pre-service teachers’ skills and competencies throughout their studies and in their professional lives. The data were collected through an online questionnaire administered 06/10/2021–22/01/2022 to first-year pre-service teachers studying at five Finnish universities. Geographically, these universities represented four regions in Finland, inlcuding a southern (*n* = 263), a central (*n* = 163), an eastern (*n* = 135), and two western (*n* = 178 and *n* = 49) universities. The response rates varied between 44% and 64% across different universities, resulting in an overall response rate of 57% (N = 788). Excluding the eleven participants who had a typographical error in their year of birth, most of the participants were aged 25 or younger (73%), and a minority was aged between 26 and 40 (18%) or over 40 (8%). The participants were in a Finnish-speaking teacher education program, with only a subset of them enrolled in a Swedish-speaking program. The participants were enrolled in three distinct teacher education programs, i.e., special teacher education (*n* = 74), early childhood teacher education (*n* = 332), and primary teacher education (*n* = 382).

### Informed consent

Ethical review and approval were not required for the study on human participants in accordance with the local legislation and institutional requirements. Before answering the questionnaire, the participants were provided with a short description of the project and its aims. This was coupled with a link to a detailed privacy notice including information on the researchers involved, contact persons, the kinds of data being collected, the ways of handling and protecting the collected data, the processing of personal data, and participants’ rights. This information was provided in accordance with the European General Data Protection Regulation. Participation in the study was voluntary, and informed consent was required of all participants at the end of the questionnaire on (1) their responses being used in research; (2) their responses being combined with the data from the student selection and study phase; and (3) their responses and the data from the student selection and study phase being combined with research data from other universities. Only respondents who gave a written consent on all three accounts were included in the study, and all data were handled without any personal information.

### Measures

#### Emotional intelligence.

The Self-Rated Emotional Intelligence Scale (SREIS) was utilised for assessing EI [16]. This scale comprises 19 items on a Likert-type scale ranging from 1 (very inaccurate) to 5 (very accurate). It encompasses 4 factors or dimensions: perceiving emotions (4 items, e.g., ‘By looking at people’s facial expressions, I recognize the emotions they are experiencing’), use of emotions (3 items, e.g., ‘When making decisions, I listen to my feelings to see if the decision feels right’), understanding emotions (4 items, e.g., ‘I could easily write a lot of synonyms for emotion words like happiness or sadness’), and managing the emotions of self and others (8 items, e.g., ‘I can handle stressful situations without getting too nervous’ and ‘I know the strategies to make or improve other people’s moods’). Previous research has demonstrated good internal consistency for the scale [16], and it has exhibited convergent validity with instruments such as the Emotional Intelligence Self-Description Inventory [35] and the Three Branch Emotional Intelligence Rating Scale Assessment [36]. In the current sample, the Cronbach's alpha values for the SREIS factors were deemed satisfactory: perceiving emotions (*α* = .63), using emotions (*α* = .74), understanding emotions (*α* = .77), and managing emotions (*α* = .73).

#### Cultural beliefs.

The Teacher Cultural Beliefs Scale (TCBS) was employed to gauge pre-service teachers’ multicultural and egalitarian beliefs [14]. This scale consists of 10 items on a Likert-type scale ranging from 1 (strongly disagree) to 5 (strongly agree). It assesses, first, pre-service teachers’ overall recognition of cultural differences, emphasising the importance of appreciating such differences and accommodating them in teaching practice (Multicultural Beliefs; 6 items, e.g., ‘Respecting other cultures is something that children should learn as early as possible’). Second, it assesses pre-service teachers’ inclination to underscore cultural similarities while emphasising the equal treatment of all students regardless of their cultural backgrounds (Egalitarian Beliefs, 4 items, e.g., ‘Children should learn that people of different cultural origins often have a lot in common’). Previous studies have substantiated the scale’s factor structure and demonstrated measurement invariance across diverse sample types. Additionally, the scale has exhibited theoretically significant connections with related constructs, such as prejudices, motivation to regulate prejudiced behaviours, attitudes toward pluralism and acculturation, and an authoritarian teaching style [14]. In the current sample, the Cronbach's alpha values for the TCBS subscales were found to be good: Multicultural Beliefs (*α* = .81) and Egalitarian Beliefs (*α *= .79).

### Statistical analysis

Using the statistical software Mplus version 8.8 [37], we first examined whether the assumed original factorial structures of SREIS and TCBS would apply to the data. Utilising Confirmatory Factor Analysis (CFA), we estimated two measurement models based on the SREIS data, one with four first-order correlated factors and another with a second-order factor. Our hypothesis was that the factor structure would exhibit four distinct, yet highly correlated, EI factors (i.e., perceiving, using, understanding, and managing emotions). We expected that each item would be represented by its own respective latent factor. By testing the second-order factor structure, we examined whether there is a latent second-order factor (i.e., general EI). Next, we used CFA to examine the factor structure of the TCBS, expecting the items to load onto two factors, i.e., Multicultural Beliefs and Egalitarian Beliefs.

We proceeded to analyse the associations between the SREIS and TCBS factors using a linear regression model to explore the relationship between them. In this regression model, we investigated whether the latent second-order EI factor predicted each of the TCBS latent variables when introduced simultaneously into the model. To refine the analysis, we designated specific factors to the residuals of the EI latent factors within the linear regression model. This enabled us to assess whether each EI factor independently predicted multicultural beliefs and egalitarian beliefs, beyond the prediction made by the latent second-order EI factor.

Before conducting the Mplus analyses, we removed the data of eleven participants who had large amounts of missing data, obtaining a sample size of 777. Because TCBS variables were negatively skewed with a longer tail on the left side of the distribution and a high concentration of values closer to the maximum values (i.e., four or five), TCBS variables were defined as categorical variables. Consequently, the models were estimated using the weighted least square means and variance adjusted (WLSMV) estimator. The WLSMV estimator has also been demonstrated to provide less biased estimates for factor loadings with categorical variables [38], providing additional support for its application.

The model fit was evaluated using root mean square error of approximation (RMSEA), comparative fit index (CFI), Tucker-Lewis index (TLI), and standardised root mean square residual (SRMR). A model fits the data well when RMSEA is lower than.06, CFI and TLI is greater than.95, and SRMR is lower than .08 [39]. We disregarded the significance of the p-value in the χ^2^ test due to the influence of our large sample size on the reliability of this indicator [40]. When evaluating the fit of the model, we also examined residual correlations, which were found to be normally distributed with a mean value 0.00 and standard deviation.051, providing further evidence that the model fit the data well.

## Results

### Descriptive statistics

Table 1 displays the descriptive findings, showing that the pre-service teachers overall reported moderate to relatively high levels of EI. The highest scores were observed in the domain of ‘perceiving emotions’, indicating a strong self-reported skill in interpreting nonverbal cues like body language and facial expressions. Conversely, ‘understanding emotions’ showed comparatively lower average levels, suggesting less confidence in understanding the complexities of emotions and their subtle differences. Additionally, the average levels of Multicultural Beliefs and Egalitarian Beliefs were notably high, nearly reaching the maximum value of ‘five’ for multiculturalism.

**Table 1:** Means and standard deviations of study variables and one-way analysis of variance between teacher education programmes.

Scale/ Subscale	Min	Max	Mean (SD) All	Mean (SD) SE	Mean (SD) PST	Mean (SD) ECE	F (df)	*p*
*Emotional intelligence*								
Perceiving emotions	1.75	5.00	4.03 (.50)	4.10 (.50)	4.05 (.48)	3.99 (.53)	2.42 (2, 784)	.090
Using emotions	1.00	5.00	3.78 (.75)	3.69 (.82)	3.76 (.76)	3.83 (.71)	1.23 (2,784)	.277
Understanding emotions	1.50	5.00	3.57 (.69)	3.66 (.65)	3.55 (.69)	3.56 (.71)	.805 (2,784)	.447
Managing emotions	1.63	5.00	3.75 (.53)	3.83 (.60)	3.77 (.53)	3.72 (.51)	1.81 (2,784)	.164
*Cultural beliefs*								
Multiculturalism	2.33	5.00	4.73 (.37)	4.81 (.22)	4.71 (.36)	4.72 (.40)	2.22 (2,785)	.109
Egalitarianism	2.00	5.00	4.38 (.62)	4.40 (.55)	4.35 (.60)	4.40 (.65)	.662 (2,785)	.516

The responses ranged between 1 and 5. SE, special teacher education; PST, primary school teacher education; ECE, early childhood teacher education.

Table 1 also presents the average levels of study variables across the three teacher education programs, i.e., special, primary, and early childhood teacher education programs. The observed differences between these groups were minimal and, upon testing with one-way analysis of variance, did not attain statistical significance. Hence, we conducted all statistical analyses using a single combined sample.

### Confirmatory Factor Analysis for the emotional intelligence and cultural beliefs scales

To examine the factorial structure of SREIS, a measurement model was specified consisting of four factors (i.e., perceiving, using, understanding, and managing emotions), each containing 3–8 original items. The four factors were allowed to correlate. The model fit was inadequate (χ^2^(98)=645.12, *p* < .001, RMSEA = .085, CFI = .925, TLI = .908, and SRMR = .057). Due to substantial modification indices between residuals, we allowed the residual covariances to be freely estimated. Additionally, three items expected to load onto the latent factor of ‘managing emotions’ displayed low factor loadings (below .24) and were consequently removed from the model. These adjustments notably improved the model fit (χ^2^(96)=323.00, p < .001, RMSEA = .055, CFI = .969, TLI = .961, and SRMR = .039).

The four EI factors correlated highly with each other, and a second-order factor was assumed to account for the correlations between the four first-order factors. When adding the second-order factor, the model fit the data equally well (χ^2^(98)=339.44, p < .001, RMSEA = .056, CFI = .967, TLI = .959, and SRMR = .042) compared to the freely estimated four-factor model. We chose the second-order factor model with four first-order factors as a final model, as it provided a good fit with the data and was more parsimonious than the model with four factors that correlated.

We proceeded to examine the factorial structure of TCBS by specifying a measurement model consisting of two factors, Multicultural Beliefs and Egalitarian Beliefs, which included 6 and 4 original items, respectively. The two factors were allowed to correlate. The model fit was unsatisfactory (χ^2^(34)=186.28, p < .001, RMSEA = .076, CFI = .974, TLI = .965, and SRMR = .051). Because of large modification indices between residuals, two covariances were allowed to freely estimate. This adjustment resulted in improved fit indices and a satisfactory model fit (χ^2^(32)=94.80, p < .001, RMSEA = .050, CFI = .989, TLI = .985, and SRMR = .037).

### Emotional intelligence predicting cultural beliefs: Multiple linear regression

We used a structural equation model to analyse the associations between the SREIS and TCBS factors. This involved employing a multiple linear regression model to investigate whether the latent second-order EI factor, together with the specific factors representing unique variance from EI factors, predicted each of the TCBS latent variables when simultaneously introduced into the model. The model fit the data well (χ^2^(286)=558.77, p < .001, RMSEA = .035, CFI = .976, TLI = .973, and SRMR = .043).

**Fig 1:**
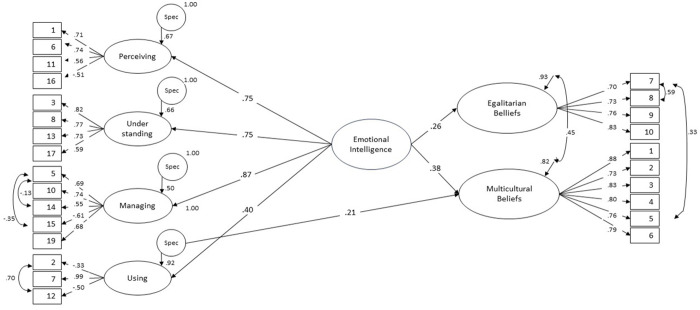
Emotional intelligence (SREIS) Predicting Cultural Beliefs (TCBS). All factor loadings and standardised regression estimates are significant at p < .001.

The regression analyses revealed that the latent second-order EI factor positively predicted the latent factors for both Multicultural Beliefs and Egalitarian Beliefs (see Fig 1), explaining 14.1% of the variance in the former and 6.8% in the latter. These relationships exhibited moderate strength, with slightly stronger associations found for multicultural beliefs in comparison to egalitarian ones. The findings indicated that higher levels of EI tended to coincide with stronger inclinations toward appreciation and accommodation of cultural differences than toward beliefs about equal treatment regardless of cultural background.

The model included specific factors that were added to the residuals of the latent EI factors within the linear regression model. This was done to enable the assessment of whether each EI factor independently predicted Multicultural Beliefs and Egalitarian Beliefs, beyond the prediction made by the latent second-order EI factor. The findings showed that the specific factor for ‘using emotions’ significantly and positively predicted Multicultural Beliefs, suggesting that the use of emotions to guide thinking and behaviour significantly predicted beliefs appreciating cultural diversity.

Finally, it was found that Multicultural Beliefs and Egalitarian Beliefs exhibited moderate correlation (.45). This finding indicated that individuals embracing multicultural values also tend to have a greater inclination toward egalitarian principles.

## Discussion

This study examined the association between emotional intelligence (EI) and cultural beliefs, using self-reported data collected from pre-service teachers at the beginning of their studies in five Finnish universities. Our analysis confirmed our hypothesis that EI would be positively associated with cultural beliefs; the latent second-order EI factor positively predicted the latent factors for both multicultural and egalitarian beliefs. Moreover, the relationships between the constructs exhibited moderate effect sizes, where multicultural beliefs had a slightly stronger association than egalitarian beliefs. Higher levels of EI tended to coincide with stronger inclinations toward appreciation and accommodation of cultural differences (i.e., multicultural beliefs) than toward beliefs about equal treatment regardless of cultural background (i.e., egalitarian beliefs).

This study suggests that Finnish student selection for teacher education, which assesses applicants’ cognitive and non-cognitive abilities, attracts pre-service teachers who exhibit readiness to develop as emotionally and culturally responsive teachers. Our study revealed that the pre-service teachers held highly positive beliefs toward cultural diversity, endorsing not just equal treatment regardless of cultural background but also expressing strong support for multicultural beliefs that appreciate cultural diversity. Moreover, our findings indicated that pre-service teachers attained relatively high scores on EI, particularly in the domain of ‘perceiving emotions’. This domain is a foundational ability involving the recognition of emotions from facial expressions, body language, and nonverbal cues. Conversely, the most demanding EI ability appeared to be ‘understanding emotions’, which encompasses the skills to grasp, accurately label, and comprehend the origins of emotions and their interconnections. Further, the EI ability ‘using emotions’ was found to significantly and positively predict multicultural beliefs, suggesting that the use of emotions to guide thinking significantly predicted beliefs appreciating cultural diversity. These findings are significant considering how positive cultural beliefs inform culturally sensitive teaching practices that value and embrace pupil diversity [1,41]. Further, they support the need to include an evaluation of non-cognitive skills in student selection measures for teacher education programmes.

The association between cultural beliefs and EI (i.e., perceiving, using, understanding, and managing emotions) contributes to the broad literature on teachers’ cultural beliefs. The association between multicultural beliefs and all EI abilities supports other key research on the topic [17,19], which reported a positive association between EI and teachers’ multicultural attitudes. Although this study did not examine implicit attitudes towards ethnic minority students as such, like other research [5], respondents’ high scoring on multicultural beliefs suggests such implicit attitudes may be positive. The average levels of Multicultural Beliefs and Egalitarian Beliefs were notably high, nearly reaching the maximum value for Multicultural Beliefs. A strong orientation to multiculturalism coupled with respondents’ moderate to relatively high levels of EI suggests that the examined cohort of pre-service teachers were pro-social, empathetic, and skilled in interactions [16,33,34], including interactions with students who have an immigrant background.

The association between Egalitarian Beliefs and EI abilities suggests the examined cohort’s cultural beliefs were enriched with the values of equality and students’ similar treatment irrespective of ethnic and cultural background. Notwithstanding, colourblind ideals and ideologies in relation to EI should be reflected on during teacher education to ensure pre-service teachers develop into critical and thoughtful material designers and educators of immigrant students [1]. This would additionally help pre-service teachers be mindful of how the habitual patterns in their judgments about (immigrant) student behaviour are formed and how such patterns contribute to the repeated experience of certain emotions, which could undermine their resilience [28]. Teacher education programs could support such reflection through diversity training, opportunities for emotion-sensitive mentoring and culturally sensitive field experiences, short interventions, and EI-focused workshops.

The findings further highlight the association between Multicultural Beliefs and the latent EI factor ‘using emotions’. That is, the examined sample of pre-service teachers were individuals who believed cultural differences are enriching and belong to a classroom environment [14]. They also considered themselves able to accurately perceive and recognize emotions, understand the complexity of emotions, regulate emotions and, particularly, make use of emotions to facilitate thinking [13]. ‘Using emotions’ is an important EI ability bearing on how emotions inform cognitive processes [42], with implications for problem solving and decision making. The association between respondents’ being proponents of multiculturalism and their reported ability to use emotions might be explained by respondents being at the very beginning of their teacher education.

Pre-service teachers who have not yet had their first teaching practice might be overly certain in their ability to control and utilize emotion in teaching situations, especially for the benefit of inclusive instructional practices. Thus, the association between Multicultural Beliefs and the EI factor ‘using emotions’ might change at a later point in teacher education depending on, for example, how aware pre-service teachers are of their attitudes toward racial minority students [24]; how pre-service teachers regard students with an immigrant background after having gained some teaching experience in a multicultural classroom [43]; or how prepared pre-service teachers feel to approach multicultural education with intelligence, professionalism, and sensitivity upon completing their initial teacher education [44]. Teacher education can take such parameters into account by incorporating group assignments with a strong introspective orientation; developing mentorship programs offering guidance and feedback from teachers experienced in diverse classrooms; as well as providing early and ongoing exposure to multicultural instructional settings in universities and schools.

The influence of emotions on beliefs is twofold. Emotions “may give rise to beliefs where none existed, or change existing beliefs; and they may enhance or decrease the strength with which a belief is held” [45, p. 45]. Thus, it could be argued that the high EI ability observed in the examined pre-service teachers might help them navigate and evaluate their cultural beliefs during teacher education. In practice, this may affect diversity-related burnout and immigration-related self-efficacy, which have been found to respectively decrease and increase when teachers perceive immigrant students as an asset instead of a problem [43]. Yet, having and maintaining strong multicultural beliefs in times when beliefs towards diversity are influenced by prejudicial policies goes beyond teachers’ beliefs in their ability to teach in culturally diverse classrooms. It affects teachers’ ability to act as agents of transformation of dominant ideologies and sustain schools as spaces for democratic resistance [46], thus potentially reducing racial or ethnic otherness in education [4].

Teachers have been found to play a significant role in students’ racial-ethnic discrimination in schools with adverse effects on student well-being and academic outcomes, which highlights the need to better educate pre-service teachers on equitable and non-discriminatory ways of teaching [47]. Such implications for teaching further stress the importance of pre-service teachers entering ITE with good EI skills and positive cultural beliefs, which can be enhanced and developed as pre-service teachers learn more about emotional understanding, teacher-student relationships, and their own moral purposes and identity as teachers [28]. At the same time, however, these implications underscore the need for ITE to offer pre-service teachers more opportunities for engagement with and reflection on more socio-cultural contexts that could potentially strengthen their multicultural beliefs, especially because the properties of emotions and the conditions under which emotions arise can influence beliefs [45]. Such opportunities might also help build pre-service teachers’ EI as a bolster against public debate contesting national multicultural values. This is pertinent to international ITE contexts, including Finnish ITE, where democracy, democratic values, and the teacher as a societal agent are generally regarded as central principles but, in practice, are unsystematically addressed [48].

This study is not without its limitations, as it drew on self-reported survey data. Pre-service teachers’ responses on EI and cultural beliefs may therefore have included a social desirability bias, meaning their skills and beliefs may in fact not be as favourable or accurate as they appear. The examined cohort may have consciously or unconsciously inflated their reported competencies and openness to align with the perceived value teacher education places on EI and cultural sensitivity. Other difficulties with self-reported data include the risks of participants misunderstanding or misinterpreting questions, participants’ tendency to respond in a particular way (e.g., always agreeing or choosing extreme options), or participants’ lack of sufficient self-awareness to understand their own behaviours and thoughts [49]. Such risks can lead to inaccurate responses. The difficulty arising from such risks was partially mitigated in this study by the use of questionnaires previously reported to have acceptable reliability and validity.

It is important to additionally note the ethical concerns in asking pre-service teachers to evaluate their multicultural or egalitarian beliefs. These issues include the risk of defensiveness, as such questions may make participants feel judged or scrutinised, especially if cultural beliefs are deeply personal or if participants feel pressure to conform to perceived institutional expectations in ITE. Although the questionnaires were administered with participants’ privacy in mind, some participants with certain beliefs may have feared being negatively labelled or judged for their perspectives, for instance favoring egalitarianism rather than multiculturalism. Self-reported surveys lack the relative objectivity of observational or performance-based assessments, raising questions about whether these self-perceptions translate into actual behaviours or attitudes in real-world teaching contexts. Therefore, although we controlled for the ceiling effect observed in the data on cultural beliefs, the relationship between these two examined constructs could be further explored qualitatively, helping to highlight nuances in pre-service teachers’ understandings.

Other study limitations concern the analysis. Our analysis with this sample of pre-service teachers suggested that multicultural and egalitarian beliefs exhibited some overlap, but we did not construct a latent second-order factor for cultural beliefs because we wanted to make visible the potentially different associations between EI and cultural beliefs. Future studies could additionally examine whether multicultural and egalitarian beliefs are empirically separate factors [14,22]. Moreover, despite the encouraging findings on the cultural beliefs of pre-service teachers still very early in their studies, it is worth noting that such beliefs should be reevaluated as these teachers gain more experience. For instance, results from teachers with more years of teaching suggest worse results towards multiculturalism outside the classroom [46]. Finally, given that beliefs are considered a major source of influence over one’s emotions [7], future research could examine the influence of pre-service teachers’ cultural beliefs on their EI skills. A longitudinal research design would help address whether and how cultural beliefs change as a result of teacher education studies, as well as whether the association of pre-service teachers’ EI with cultural beliefs is sustained over time. In spite of these limitations, our study contributes to the paucity of literature exploring emotional, social, and intercultural competencies simultaneously [12], as well as to the scarcity of empirical studies on pre-service teachers’ EI skills and multicultural attitudes [17].

## Conclusions

This study suggests that the admission process for ITE in the examined context selected individuals who already possessed the crucial abilities of EI and cultural responsiveness necessary for effective teaching. Multicultural and egalitarian beliefs exhibited a moderate correlation (.45), suggesting that pre-service teachers embracing multicultural values might also have a greater inclination toward egalitarian principles. This is an important finding for Finland, where pre-service teachers have reported being undereducated on topics pertaining to human rights and equality, while the country prides itself on championing such principles (e.g., national curricula) despite persisting inequalities in education [50]. This is also an important finding for ITE in general, as pre-service teachers should have favourable beliefs about multiculturalism to be able to work in schools with increasingly diverse student demographics, especially in larger urban areas. The study provides empirical evidence for the association between pre-service teachers’ EI and cultural beliefs, thus supporting the need to acknowledge emotions and cultural beliefs as integral to the teaching profession [1], as well as the need to address such matters already present in the pre-service teacher education curriculum more explicitly and consistently [48].
